# Buffalo Academy of Medicine

**Published:** 1902-05

**Authors:** Irving P. Lyon

**Affiliations:** Secretary


### SOCIETY PROCEEDINGS.

### Buffalo Academy of Medicine.

*Section on Pathology, March 18, 1902.*

Reported by IRVING P. LYON, M. D., Secretary.

THE regular monthly meeting of the section on pathology, Buffalo Academy of Medicine, was held in the Academy rooms, Public Library Building, Tuesday, March 18, at 8.30 p.m., Dr. Grover W. Wende, chairman, presiding.

I.—EXHIBITION OF PATHOLOGICAL SPECIMENS.

1. Dr. Herbert U. Williams presented specimens of gastric ulcer, as follows: (*a*) A gross specimen of gastric ulcer, which was found associated with a severe grade of chronic gastric catarrh, secondary to chronic passive congestion. Dr. Williams called attention to gastric catarrh as a predisposing cause of gastric ulcer. (*b*) Microscopic section of a gastric ulcer, showing an eroded bloodvessel, with secondary thrombus formation. The thrombosis had developed too late to prevent hemorrhage and death. (*c*) Large gastric ulcer (gross specimen), showing an eroded artery with calcareous atheromatous degeneration, the local arterial lesion being part of a high grade arterio-sclerosis with atheroma but shown in the aorta. This process was emphasised as another cause of gastric ulcer.

Dr. Allen A. Jones remarked that Dr. Williams's specimens confirmed his personal belief that gastric ulcer had no single cause, but was due in different cases to different causes; in fact, to a great variety of causes.

2. Dr. Eugene A. Smith showed a specimen of thrombosis of the abdominal aorta, the thrombus extending from the bifurcation of the aorta upward to the origin of the inferior mesenteric artery. Atheroma of the abdominal aorta was not noted, though arterio-sclerosis of the ascending aorta and an old fibroid mitral stenosis were found. During life the patient had shown marked dysenteric symptoms and signs of the obliteration of the arterial circulation in the legs. Pain in the feet, extending upward into the thighs, was a prominent symptom. There had been no abdominal pain or tympanites. Vomiting and diarrhea, fever, rapid pulse, and the like, ending in collapse and death, were the chief clinical features, with the signs of obliteration of the arterial circulation in the legs. The diagnosis *intra-vitam* had been correctly made, as revealed by autopsy. Dr. Smith raised the question, whether the thrombosis was to be regarded as infective and secondary to the dysentery, or whether the dysentery was secondary to the thrombosis of the aorta, which involved the origin by the inferior mesenteric artery? He inclined to the latter view.

(3) Dr. H. R. GAYLORD presented microscopic sections of four cases of carcinoma in hogs. These four cases were all found during a period of two months in one of the large abattoirs in Buffalo. Carcinoma in swine, therefore, cannot be the rare disease it has been supposed to be by previous writers.

II.—REPORTS OF CASES.

1. *Two Cases of Tertian Malarial Fever, Originating in West Seneca, N. Y.*, by Mr. Eugene Horton (student, Medical, University of Buffalo.) Mr. Horton exhibited blood films showing tertian malarial parasites from two cases, mother and a five-year old child. These cases were important in establishing the fact that malaria may and did have a local origin in this region of the country, almost within the limits of the City of Buffalo. The mother had not been away from the region of Buffalo for many years, and the child, according to the mother's positive assertion, had never been on a railway train or left the immediate vicinity of its home. These two cases were examined by Mr. Horton about a week ago—March, 1902—and by blood examination proved to be malaria of the tertian type. Symptoms of the disease had been manifested in the child for about three weeks; in the mother for a shorter period.

Upon careful investigation and inquiry, however, the following history was obtained:

The family moved into its present residence August 7, 1901, from a house about one-half mile distant. In this former residence, during June and July, 1901, father, mother and child all had typical symptoms of malarial fever and the affection was diagnosticated and treated by the physician as malaria. The cases of the mother and child had been mild and yielded to quinine. The father's case had been resistant and continued for over two months, until they moved away into a new residence.

It was, therefore, apparent that the attacks of malaria occurring in March of the present year in the mother and child represented simply recrudescences from the previous infection of last summer, which had in the interval been quiescent but not eradicated.

Whence the infection of last summer? For we no longer believe in a miasmatic origin of malaria, but know that we must have two factors present in any locality to permit the local development of malaria — namely, mosquitoes of the genus *anopheles*, and previous cases of malaria from which the anopheles may become infected, and capable of infecting healthy people with malaria. We must, therefore, presuppose the existence of both these factors in the immediate locality of the residence of these cases.

In regard to the presence of mosquitoes, we have the statement of the mother that their former residence was swarming with these insects, and that "we were almost eaten alive by them last summer." Furthermore, there was a creek within a hundred yards of the house, a small pond in the backyard, and a large pool of stagnant foul water beneath the house, formed by the discharge of house slops under the house. This pool under the house was said to be alive with mosquitoes. It is not far to arrive at the conclusion that this pool was a breeding place for anopheles. Moreover, we know that anopheles have, though only in a few isolated instances, been found in the neighborhood of Buffalo.

Presuming, then, that the anopheles were present, can we show any previous case of malaria from which they could have become infected? That malaria is not endemic here has been abundantly shown. We must therefore assume some exceptional explanation of the possibility of the infection by the mosquitoes. This is forthcoming in the fact that a large gang of Italian laborers, employed by the Depew water works, was present in the immediate vicinity of the house last spring during the month of May for about three weeks, engaged in laying pipes within a few rods of the house. One or two of these Italians remained on guard during the night.

We have, then, a probable explanation of this local epidemic of malaria, as follows: "During the month of May, 1901, a gang of Italian laborers was employed close to the house of our patients. Malaria probably existed among these Italians, who were more than likely freshly arrived from Italy. The mosquitoes, *anopheles*, breeding in the foul collection of waters under the house, fed upon the malarial blood of these Italians, became infected with the parasites, and in turn infected the inhabitants of this house with malaria. Thus is explained in a rational and plausible way the occurrence of the first cases of malaria of recent years, positively shown to have had a strictly local origin.

DISCUSSION.

Dr. De Lancey Rochester complimented Mr. Horton on his interesting and important contribution, being the first proved case of malaria of local origin known in recent years. Dr. Osler, however, was authority for the statement that in former times malaria in endemic form was no stranger to the lower lake region. Hence, formerly at least the anopheles must have thrived here, and doubtless can now. Malaria may become endemic again at any time if it once gets a start and under headway.

Dr. Herbert U. Williams said that as the Italians had found it possible to protect themselves against malaria by mosquito-netting, oil on the breeding pools, and the like, so also it must be possible for us to continue to keep ourselves and locality free from malaria, by stamping out such local epidemics as this one. The board of health might properly be interested in such cases.

Dr. Irving P. Lyon stated that he had failed to find a single specimen of anopheles among 374 mosquitoes collected during the summer of 1900, in and about Buffalo, within a radius of about twenty miles. A local entomologist, Mr. M. F. Adams, during the same summer collected and examined between 300 and 400 mosquitoes from Buffalo and its vicinity and found three specimens of anopheles among these. Thus out of about 800 mosquitoes, 3 anopheles were found.

During the summer of 1901, Dr. Lyon examined several thousand mosquitoes, caught or bred from the larvæ obtained in the City of Buffalo, on Squaw Island, and from the neighboring village of Williamsville. Three specimens only of anopheles were found among the several thousand examined, the three anopheles all coming from the outskirts of Williamsville. Thus up to the present, we have records of only six identified specimens of anopheles found in or near Buffalo. But these six specimens are sufficient to prove the presence in this climate of the malaria-bearing mosquito. It is likely that more extensive investigation will show that the anopheles exists about here more commonly than supposed, perhaps here and there in small foci.

Dr. Lyon had seen the cases of malaria reported by Mr. Horton and had examined the blood of both, and confirmed in full the statements made by Mr. Horton. The explanation of the local epidemic seemed to be the correct one and left very little to be desired for completeness. A similar local epidemic in an inland town in Massachusetts had been lately traced to the same origin, *i.e.*, a gang of Italian laborers.

The proof of such a case or two of malaria must not be made to do service hereafter for the indiscriminate diagnosis of malaria. These cases are in every way exceptional, and are explained by exceptional circumstances. The only proper diagnosis of malaria is by blood examination.

(2) *An Unusual Case of Mediastinal Tumor Associated with Obstruction of the Thoracic Duct and Accumulation of Chyme in the Pleural Cavity*, by Dr. H. G. Matzinger. Such a case was described clinically and specimens of the milk-white fluid aspirated from the left pleural cavity were exhibited. Large quantities of this chylous fluid were aspirated on several occasions. From the right pleural cavity a clear, yellow fluid was aspirated in large amount, in nowise resembling the chylous fluid obtained from the left chest. Physical examination showed only a mass of soft glands arising from under the left clavicle at its middle portion, and a gland in the right axilla about the size of a hickory nut. These glands were supposed to be sarcomatous or lymph-adenomatous. The only explanation of the chylous fluid in the left chest was an obstruction of the thoracic duct by the new growth. The patient was without temperature, and had lost about sixty pounds in weight since he first noticed a tumor above the left clavicle about ten months ago. Urinalysis was negative. The blood examination made in the day time showed 10,000 leucocytes and no *filariæ sanguinis hominis*. No evidence of tuberculosis was discovered, although there was a history of consumption in two cousins. Careful chemical analyses of the fluid made by Dr. Clowes were presented, showing a close similarity with the chemical analyses of chyle.

Dr. Williams said that Dr. Matzinger had overlooked one possible explanation of the condition—namely, a perforation of the esophagus by an epithelioma. This was a mere suggestion and not offered as a diagnosis.

Dr. ALLEN A. JONES called attention to the extreme rarity of the case and the difficulty of explaining the chylous fluid in the left chest and the clear fluid in the right chest. There must be a perforation of the thoracic duct.

Dr. Lyon remarked that the absence of temperature made the case a favorable one for testing with tuberculin for tuberculosis. With reference to the possibility of filariasis, Calvert's recent studies on two cases have shown a marked eosinophilia in the blood. The blood should be carefully examined in this case, before a diagnosis is reached.

(3) *A Case of Very Early Syphilis of the Brain*, by Dr. J. HENRY DOWD. The following interesting case shows how early the brain may become involved during the secondary period of that disease:

X.Z., aged 28, reported for treatment on September 26, with several ulcerations on glands of penis, also discharge from the urethra, both of which appeared yesterday (25th), and positively stated that the last coitus occurred on September 1. Microscopical examination showed the discharge to be gonorrheal in nature. (Note incubation period of 25 days.) The ulcerations were cauterised and an opinion given of possible mixed infection. All the ulcerations healed except one which became angry, and on the 4th of December, 67 days after its appearance, macular eruption appeared on the abdomen, forehead and chin, but very slight, there not being over 50 spots all told.

Treatment for syphilis was instituted at once, per mouth. January 6, complains of headache, seeming all over the head, noticeable most at night; medicine increased to toleration. January 20, headache getting worse; unable to sleep. Pain mostly over right side; photophobia; cigar dropped from hand yesterday; feeling in head as though there was a something there that moved backward and forward; medication commenced by hypodermic method. February 5, on account of tenderness of gums, injections reduced in strength for past few days. Now, aphasia lasted half a day; dragging of right foot; very constipated and vomited. February 20, on account of cramps, and the like, medicine reduced in strength, head was now as bad as ever—no let up day or night; face twitches and pain is extending to the back of the neck. Priest called to see the patient, as he was thought to be dying. March 6, slight relapse on account of having to decrease medicine. Now increased and steady improvement follows. At no time did temperature exceed 99°, he has lost 40 lbs. in weight and in 40 days has taken 9⅜ grains of bichloride hypodermatically.

This case cannot be considered tertiary, as at no time were symptoms present indicating gumma, or thrombotic involvement of the bloodvessels.

Dr. A. L. Benedict read a paper entitled,

CHRONIC BASIS OF DIETETICS.

Dr. Benedict discussed briefly the subject of the chemical demands of the body and the rational supply of such demands by means of the different classes of foodstuffs.

The discussion was shared by Drs. A. A. Jones, Lyon and Clowes.

Members present, 30; adjournment, 10.45 p.m.

